# Bio-Inspired Toughening of Composites in 3D-Printing

**DOI:** 10.3390/ma13214714

**Published:** 2020-10-22

**Authors:** Johannes Stögerer, Sonja Baumgartner, Alexander Hochwallner, Jürgen Stampfl

**Affiliations:** TU Wien, Getreidemarkt 9, 1060 Vienna, Austria; johannes.stoegerer@tuwien.ac.at (J.S.); alexander.hochwallner@tuwien.ac.at (A.H.); juergen.stampfl@tuwien.ac.at (J.S.)

**Keywords:** 3D-printing, stereolithography, inkjet printing, composites, bio-inspired, fracture toughness

## Abstract

Natural materials achieve exceptional mechanical properties by relying on hierarchically structuring their internal architecture. In several marine species, layers of stiff and hard inorganic material are separated by thin compliant organic layers, giving their skeleton both stiffness and toughness. This phenomenon is fundamentally based on the periodical variation of Young’s modulus within the structure. In this study, alteration of mechanical properties is achieved through a layer-wise build-up of two different materials. A hybrid 3D-printing device combining stereolithography and inkjet printing is used for the manufacturing process. Both components used in this system, the ink for jetting and the resin for structuring by stereolithography (SLA), are acrylate-based and photo-curable. Layers of resin and ink are solidified separately using two different light sources (λ_1_ = 375 nm, λ_2_ = 455 nm). Three composite sample groups (i.e., one hybrid material, two control groups) are built. Measurements reveal an increase in fracture toughness and elongation at break of 70% and 22%, respectively, for the hybrid material compared to the control groups. Moreover, the comparison of the two control groups shows that the effect is essentially dependent on different materials being well contained within separated layers. This bio-inspired building approach increases fracture toughness of an inherently brittle matrix material.

## 1. Introduction

**Figure 1:**
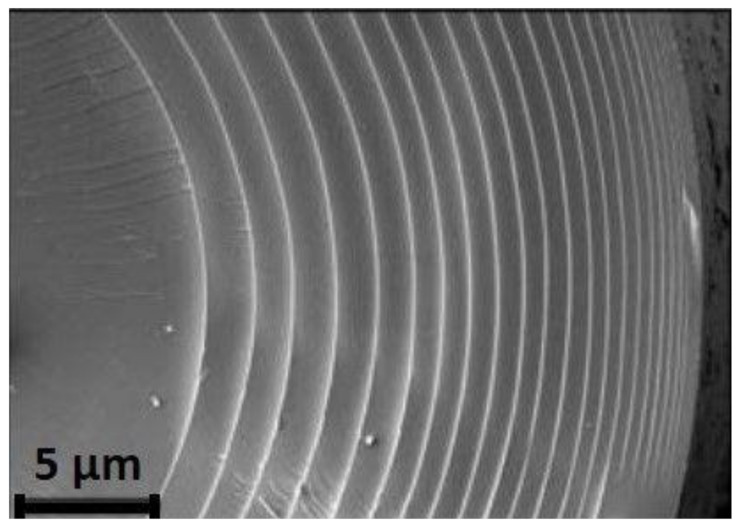
SEM of a cross-section through a typical spicule. Laminated structure composed of thin organic layers between silica layers [10]. Reprinted with permission from AAAS.

Engineering high performance parts which are strong and stiff on one hand and tough on the other is challenging. While strength and stiffness provide high load bearing capability, high toughness prohibits fracture [1]. Since they are mostly mutually exclusive, combining high values of strength, stiffness, and toughness in artificially manufactured parts is a sophisticated task [1,2,3]. In contrast, multiple examples of composites linking high hardness and high toughness can be found in nature [4,5,6,7]. Nacre found in the internal layers of mollusc shells [8,9] and the skeleton of Euplectella Aspergillum a deep-sea sponge from the Western Pacific combine hard inorganic phases with softer organic ones to gain outstanding mechanical properties [10,11]. Although the basic concept of a hierarchical structure, consisting of mainly inorganic minerals and a small fraction of organic proteins, is similar in both natural composites, and the toughening mechanisms occurring are diverse [12,13]. Nacre, its remarkable properties and its toughening mechanisms, have been studied intensively [14,15,16]. Small platelets of aragonite (i.e., CaCO_3_) are separated and connected via organic material [17,18]. The unique brick-and-mortar structure allows crack deflection and branching of cracks inside the material [19,20]. The body of Euplectella Aspergillum, a hexactinellid sponge, is composed of cylindric spicules consisting of alternating layers of silica (SiO_2_) and organic layers [21], depicted in Figure 1. 

With silica layers being 0.1–0.2 µm thick and organic layers being in the order of ca. 5–10 nm, the volume fraction of the organic phase is typically about 1% [21,22,23]. This lamellar organization gives the body strength and stiffness as well as toughness [7,22].

The underlying phenomenon providing these exceptional mechanical properties is called shielding effect [24]. A crack propagating through the material is arrested inside a softer layer and further spreading is hindered or prevented [25]. The shielding effect is critically dependent on unequal mechanical properties (i.e., Young’s modulus E and yield strength σ_y_) of soft and hard materials utilized to construct a part [26,27]. For effective shielding inside the softer phase, the ratio of elastic moduli has to exceed five [26]. Computational analysis suggests that even very thin layers of soft material are capable of effective crack shielding [26,28]. Thus, the loss in stiffness of the structure is minimal compared to the homogenous part [29,30]. Therefore, it is reasonable to construct specimens with numerous soft interlayers which hinder crack propagation without corrupting other mechanical properties. Multiple sources of crack shielding in a single structure can be achieved by designing samples with continuously alternating material layers, each reducing crack propagation velocity and potentially preventing further crack spreading completely [23,31]. This general concept is valid in ceramic composites as well as brittle polymer layers combined by adhesive bonds [30].

In 3D-printing, using light-curable photopolymers based on acrylates and methacrylates fracture toughness of produced parts is a fundamental problem [32,33]. Although these substances provide high spatial resolution and good curing, end products are inherently brittle [32]. Different approaches to tackle toughness in 3D-printed products have been taken [34,35,36]. Recent approaches focus on the incorporation of inorganic particles [37], liquid or solid rubbers (i.e., Core–Shell particles) [38,39], and self-assembly of block copolymers [40]. Using photopolymers, 3D-printing technology allows to produce parts in a layer-by-layer process with exceptional resolution [41,42]. The thickness of single layers can be reduced to the range of single micrometers or even nanometers [35,43]. Additionally, almost any geometry can be fabricated [44]. In various industrial sectors (e.g., automotive manufacturers, aerospace companies, medical industry) the combination of outstanding spatial resolution and almost arbitrary geometry design is utilized to produce high quality components [45]. 

In recent studies, the shielding effect was implemented using 3D-inkjet printing [46,47]. In both studies, fracture toughness was improved by printing a single thin layer of compliant material into the stiff material. However, material selection, especially in particle-filled batches, is limited because of potential printhead nozzle clogging [48]. Other studies verified the shielding effect using co-extrusion processes to produce polymer composite materials [27,49]. Nevertheless, only very few layers of compliant material were used. Furthermore, the achieved layer-thickness was approximately 35 µm because of limited spatial resolution of the selected method. Stereolithography (SLA) is an approved high precision 3D-printing method for the manufacturing of composites [44,50]. In the current study, a hybrid system using SLA and direct inkjet printing was utilized to build ceramic composite specimens consisting of two different materials. This system provides high resolution and continuous alteration of materials with each layer. Thus, the basic construction concept of Euplectella Aspergillum could be mimicked in a reasonable manner. 

## 2. Materials and Methods 

### 2.1. Hybrid 3D-Printing System

This study was conducted using a hybrid printing process developed at TU Wien [51,52]. This system combines SLA, based on the principal of digital light processing (DLP), and direct inkjet printing in the same process. The liquid resin was contained in a transparent rotatable material vat, which allows compact machine design. The building platform was immersed into the resin. In a first step, a digital mirror device (DMD) projected UV-light (wavelength λ = 375 nm) through the vat and cured the resin in a layer-wise process, thus building a three-dimensional matrix. After the solidification of each layer, the building platform with the adhering building part was lifted from the material bath and the vat was coated with a fresh layer of liquid resin. The coating process was performed via the combination of the rotatable vat and a coating knife. This system ensures very thin liquid resin layers in the range of sample layer thickness and good circulation of the resin. Thus, undesired residues adhering to the samples are drastically reduced. Moreover, the printing process was accelerated and more viscous resins of up to 20 Pa·s were processable. The light engine (Luxbeam 4600, Visitech Engineering GmbH, Wetzlar, Germany) provided a spatial resolution of 50 µm in horizontal axes. Minimal layer thickness is theoretically around 10 µm using this system. However, due to the powder particle size, reproducible results with the materials present were only feasible at a minimal thickness of 25 µm. The penetration depth for the used composite was 180 µm at 25 mW/cm^2^ for 4 s, thus multiple times the layer thickness, to ensure sufficient curing of the previous layers. Standard Triangle Language (STL) files were converted to a PNG-file for each layer which contain the necessary information for the light exposure of each individual voxel.

**Figure 2:**
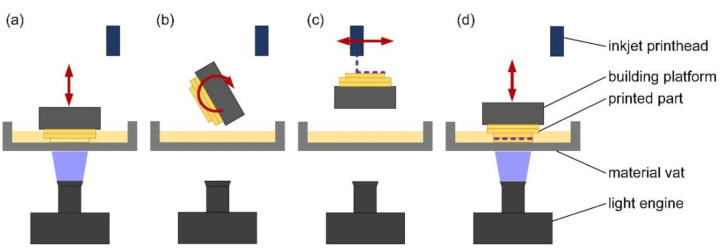
Workflow of the hybrid printing system. (**a)** The building platform immerses into the vat and a resin layer is printed; (**b**) the building platform rotates upwards; (**c**) an inkjet layer is printed and fixated; (**d**) after rotating downwards, the ink layer is fully cured and the next resin layer is printed.

In a second step, after the solidification of one resin layer, the building platform with the adhering matrix rotated upwards. In this position, a thin film of ink was applied via an inkjet print head (Xaar 1003 GS40, Xaar, UK). The print head provides a spatial resolution of 360 dpi, which is two rows of nozzles with 180 dpi each and a distance of approximately 141 µm between nozzles of one row. This print head has a nozzle diameter of 48 µm. The device operates in single pass mode with 70.5 µm between adjoining drops and 40 pL drop volume for the present ink. The application of ink was managed via bitmap-files of according resolution. Within the inkjet system, the ink was circulating permanently, thus prohibiting any phase separation. Moreover, nozzle clogging was prevented by completely omitting particles in the ink phase. In a third step, the liquid ink was fixated to the solidified resin layer by exposition to a light emitting diode (LED) array (λ = 455 nm) under nitrogen atmosphere to reduce oxygen inhibition. The array provided an exposure dose of 44 mW/cm^2^ and a penetration depth of 200 µm. In the next SLA curing step, the ink layer adhering to the already solidified layers as well as the next layer of resin were fully cured. These steps were repeated until the building process was completed. A full working cycle is displayed schematically in Figure 2.

### 2.2. Material Composition and Preparation

Two different liquid material compositions were mixed at room temperature using a Speed Mixer™ (DAC 150FVZ, Hauschild and Co. KG, Hamm, Germany). For the filled resin, organic substances were filled into a container and mixed for 10 min at a speed of 3500 rotations per minute. This ensured a homogenous batch. Thereafter, tricalcium phosphate (TCP) and the rheology additive were added. Particle size distribution measurement (Mastersizer 2000, Malvern Panalytical, Malvern, UK) revealed a mass-median-diameter of 6.88 µm for the present TCP batch. To ensure a homogenous resin, all components were mixed for 10 min at a speed of 3500 rotations per minute. The resin masterbatch has a total mass of 200 g and a viscosity of 10 Pa_·_s at 23 °C. All ink components were put in a container and mixed for 10 min at a speed of 3500 rotations per minute to create a homogenous batch. The ink batch has a total mass of 30 g and a viscosity of 10 mPa_·_s at 23 °C. After the mixing process, both resin and ink were degassed separately in a vacuum chamber for 10 min. Compositions for resin and ink are displayed in Table 1.

Two different photoinitiators were utilized to ensure a proper printing cycle. TPO-L, a constituent of the resin, absorbs solely in the wavelength range of 370–410 nm wavelength, thus is not affected by the LED array. Phenylbis (2,4,6-trimethylbenzoyl)-phosphine oxide BAPO offers a broader absorbance spectrum, thus allowing the fixation via the LED array and the complete curing with the SLA light engine [53].

### 2.3. Sample Preparation 

Three testing groups with different compositions were prepared. For the first group (A), the inkjet system was omitted and samples solely consisting of resin were built. The second group (B) consisted of a blended batch of resin and ink. The specimens for group B were built solely with the DLP system utilizing the material in the vat. For the third group (C), alternating layers of resin and ink were solidified separately. Table 2 shows the compositions and systems used for the manufacturing of each group.

For each group, three different types of testing samples were manufactured in the same 3D-printing machine, utilizing a single master batch for resin and ink, respectively. Via a heating device positioned underneath the vat, vat surface temperature was held constantly at 55 °C to reduce viscosity and ensure sufficient coating. Measured layer height was 25 µm for pure DLP-printed layers and five to eight micrometers for pure ink layers. All specimens were manufactured in XYZ orientation according to DIN EN ISO/ASTM 52921, where the biggest sample area was adhering to the building platform. After the printing process, all samples were cleaned manually using paper tissues. Thereafter, cleaned samples were post-cured for 10 min using a UV floodlight (Intelliray 600, Uvitron, West Springfield, MA, USA).

Dynstat specimens for fracture toughness measurements were constructed according to DIN 53435, which was applicable for small sample dimensions and batch sizes. More commonly used impact strength tests (i.e., Izod DIN EN ISO 180 and Charpy DIN EN ISO 179-1) [54] were not feasible due to limited building space on the building platform. Samples for bending tests and dynamical mechanical analysis (DMA) were built according to DIN EN ISO 178 and DIN EN ISO 6721-1, respectively. Overall layer thickness for all samples was 25 µm. This was given by the printing process and controlled through the 3D-printer. Resin layer thickness for groups A and B solely manufactured by DLP was 25 µm. In group C samples, an inkjet layer was added. However, the overall sample layer thickness remained 25 µm. Thus, resin layers in group C samples were thinner than in groups A and B, reduced by the thickness of the ink layer. The latter can be calculated for each sample geometry using the print head resolution of 360 dpi, the drop volume of 40 pl, and ink density of 1.1 g/cm^3^. Calculated values for sample dimensions, number of layers to gain the respective height, and layer thickness for all test specimens and groups are summarized in Table 3.

After manufacturing, all samples are sanded and stored in a desiccator according to ISO 291.

### 2.4. Testing Procedure 

**Figure 3:**
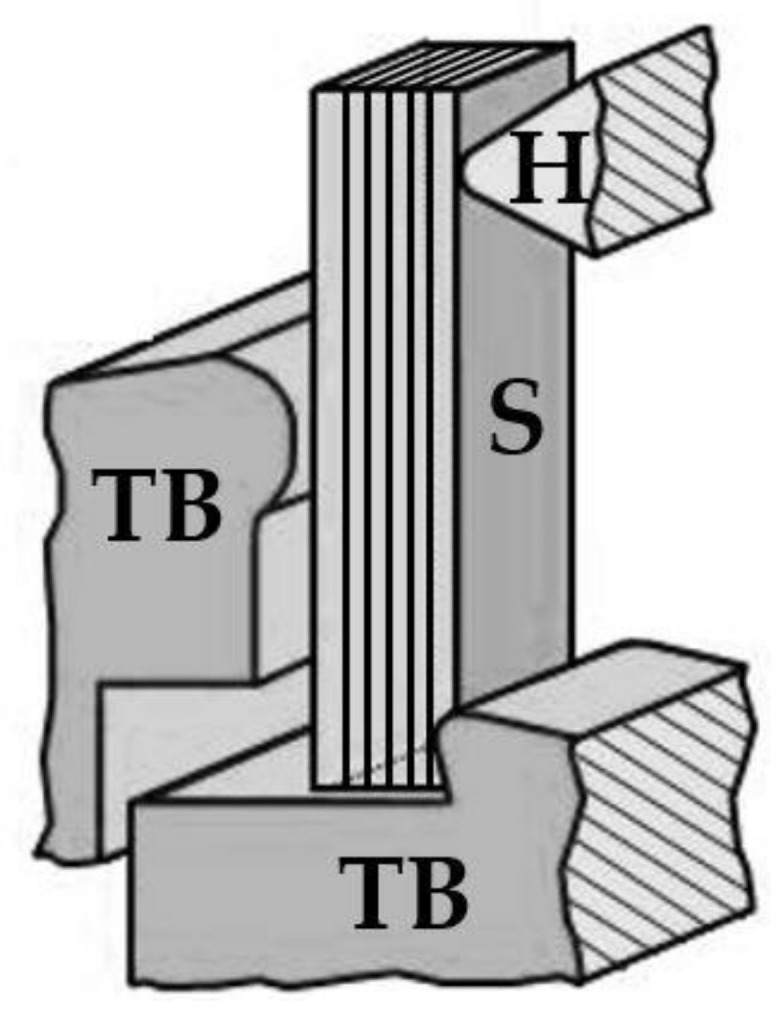
Schematic Dynstat impact strength test set-up for an unnotched sample (S) fixed between two thrust blocks (TB) and punched by a hammer (H). The applied force is perpendicular to the layer interfaces, adapted from [55].

All mechanical tests were conducted within one day to ensure constant testing conditions (i.e., room temperature, humidity). Unnotched Dynstat specimens were tested using a pendulum impact tester Frank 573 (Karl Frank GmbH, Weinheim, Germany) with a 0.5 J (5 kp cm) hammer attached. Sample orientation was selected with respect to the trajectory of the hammer fin. This specific set-up ensured that direction of applied force and layer interfaces were perpendicular, thus guaranteeing crack propagation through various layers. The experimental set-up is depicted schematically in Figure 3.

For this set-up to yield correct data, it was essential for the hammer to fully punch through the test sample. The energy required to split the specimen fixed in the apparatus was equal to the absorbed energy, which was the fracture toughness of the specimen. This value was directly provided by the testing device. Eight Dynstat samples of each group were tested.

Bending tests were conducted in a 3-point bending set-up. All samples were tested in a universal testing machine type Zwick Z050 (ZwickRoell, Ulm, Germany), until fracture occurred. The corresponding software testXpertII (Version 3.6, ZwickRoell, Ulm, Germany) was used to analyze the results. A deformation rate of 200 mm/min and a preloading of 0.1 MPa were chosen to perform all bending tests. Yield strength and edge fiber extension at this instant were measured as well as Young’s modulus and elongation at break. Ten samples of each group were tested.

The investigation of the temperature dependence of elastic properties was conducted via the DMA. A DMA 2980 Dynamic Mechanical Analysis testing device (TA Instruments, New Castle, DE, USA) and the testing software Universal Analysis 2000 (TA Instruments, New Castle, DE, USA) were used for sample analyzation. All DMA measurements were conducted in a 3-point bending setup. A temperature range from −50 °C to 110 °C was evaluated. First, samples were cooled to −50 °C using liquid nitrogen. Second, sample temperature was held constant at −50 °C for 5 min to ensure homogeneous temperature distribution in all sample regions. Third, the samples were heated up to 110 °C with a chosen heating rate of 3 °C per minute. All samples were preloaded with 0.1 N and examined at a constant frequency and amplitude of 1 Hz and 20 µm, respectively. DMA provided valuable information about the material behavior over a wide range of temperature. Parameters achieved were storage modulus, closely connected to the Young’s modulus, loss modulus representing the dissipated energy, and the dissipation factor which was connected to both storage and loss modulus, indicating the glass transition temperature of the sample [56]. 

Polymers showed viscoelastic behavior, that is, structural characteristics related both to fluids and to solids. Viscoelasticity strongly depends on the ambient temperature. Knowledge about application range and temperature for a specific polymer material is imperative. The glass transition temperature characterizes the transition of a polymer from a stiff and brittle into a viscous and compliant state, thus limiting the application range of load bearing components. Obviously, an increase in impact strength via the introduction of a softer component influences the glass transition temperature of a given specimen. Moreover, the overall storage modulus, closely linked to the stiffness, was reduced through compliant material layers. Three samples of each group are tested.

Optical analysis of the specimens’ structure and layer-wise build-up was conducted using a digital microscope VHX-6000 (Keyence, Osaka, Japan). Separate layers of group C Dynstat specimens were qualitatively verified. 

Further quantitative analysis of the different material layers was conducted using a scanning electron microscope (SEM) XL 30 SEM (Philips, Andover, MA, USA) in combination with an energy-dispersive X-ray spectroscope (EDS) Element EDS System (EDAX, Mahwah, NJ, USA). 

## 3. Results

Preliminary bending tests with specimens solely consisting of resin and ink respectively show that Young’s modulus of these two materials is substantially different. Measured values of Young’s modulus for pure resin parts are 366.83 MPa ± 45.12 MPa and 5.43 MPa ± 0.89 MPa for pure ink parts. Thus, the Young’s modulus ratio for these materials is about 67 and the primary design criterion for damage-tolerant materials via the introduction of compliant material layers is met. Ten samples (i.e., five pure resin and five pure ink samples) used for these tests are produced separately to ensure that the prerequisites are met and do not belong to the testing groups A, B, and C.

### 3.1. Impact Strength Tests

**Figure 4:**
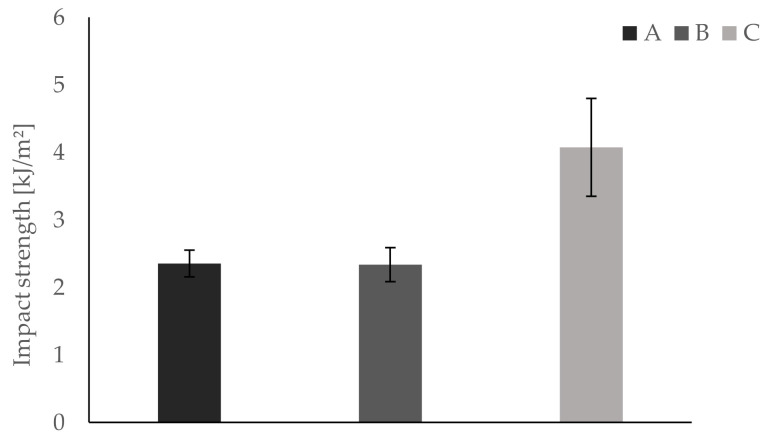
Dynstat impact strength tests show significantly higher impact strength for group C compared to groups A and B.

All testing samples are tested within three hours to ensure constant conditions (i.e., room temperature, humidity). Temperature is held constantly at 23 °C via air condition. The samples are positioned in the apparatus so that the first printed layer (i.e., the layer adhering to the building platform) is facing the direction of force application. Results show significantly higher values in Dynstat impact strength for group C compared to A and B. The measured values are 2.35 kJ/m^^2^^ ± 0.20 kJ/m^^2^^ for group A, 2.33 kJ/m^^2^^ ± 0.25 kJ/m^^2^^ for group B, and 4.07 kJ/m^^2^^ ± 0.72 kJ/m^^2^^ for group C. The results are depicted in Figure 4. 

### 3.2. Bending Tests

All bending tests are performed within five hours to ensure constant conditions (i.e., room temperature, humidity). Temperature is held constantly at 23 °C via air condition. All testing samples are fixed in the apparatus in the same way, such that the first printed layer represents the bending site of the sample. Results show differences in Young’s modulus, yield strength, and elongation at break between groups A and B on one side and group C on the other. Mean values and respective standard deviations are summarized in Table 4. 

**Figure 5:**
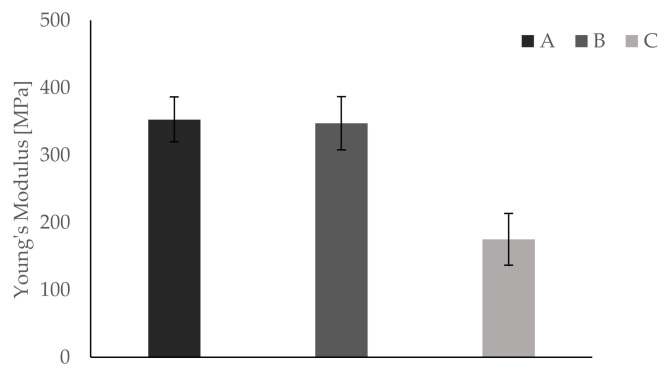
Bending tests show significantly higher values of Young’s modulus for groups A and B compared to group C.

**Figure 6:**
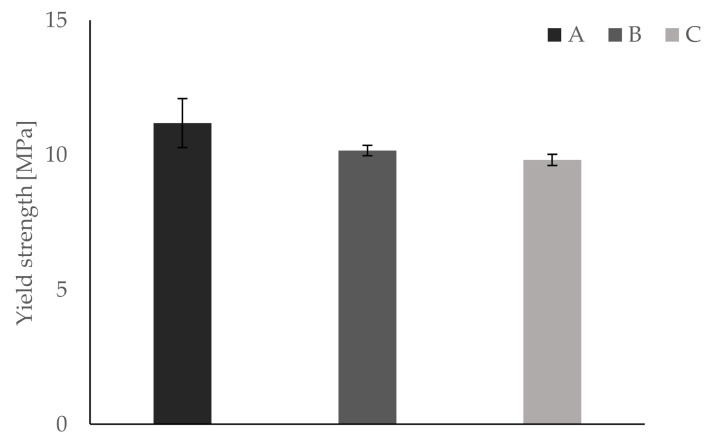
Bending tests show a reduction in yield strength for group C compared to groups A and B.

**Figure 7:**
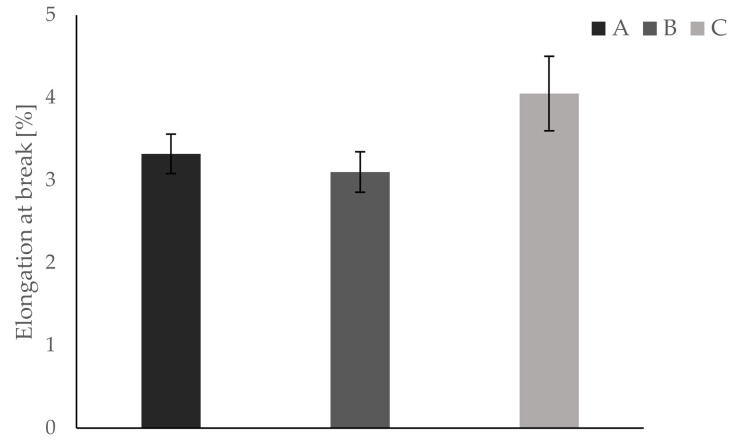
Bending tests reveal an increased elongation at break for group C compared to groups A and B (increase of >20%).

**Figure 8:**
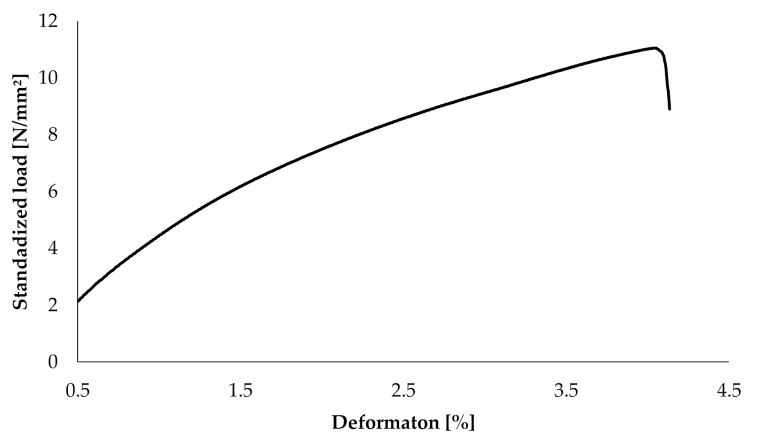
Typical load displacement curve of a group C test specimen.

Furthermore, results for each parameter are displayed separately in Figure 5, Figure 6 and Figure 7. In Figure 8, a typical load deformation curve for a group C specimen is displayed. 

### 3.3. Dynamic-Mechanical Analysis

**Figure 9:**
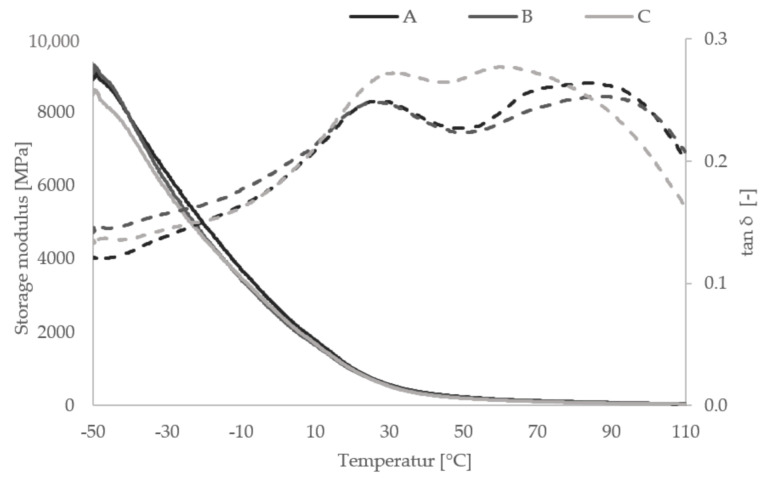
Results of the dynamical mechanical analysis (DMA) measurements. Means of storage moduli (continuous line) and tan δ (dashed line) for groups A, B, and C.

The extend of alteration induced by different mechanical property materials in one sample is analyzed using DMA. Mean curves of storage modulus and tan δ for all groups are depicted in Figure 9.

### 3.4. Imaging Analysis

**Figure 10:**
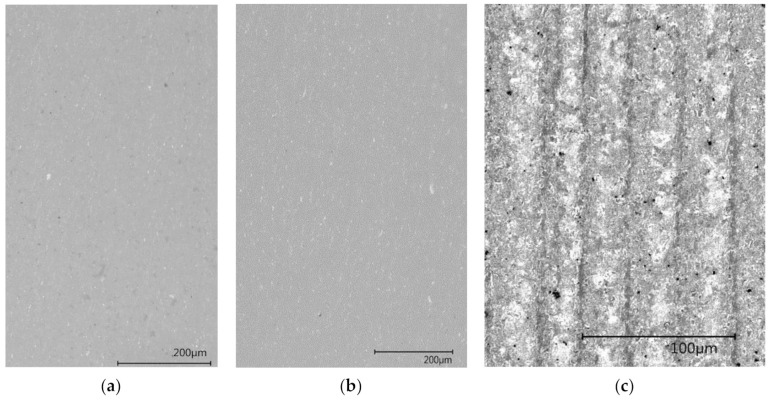
Digital microscopy images reveal homogenous structures for group A samples (**a**) and group B samples (**b**). Resin layers separated by ink layers are visible for group C samples (**c**).

Digital microscope imaging reveals remarkable differences in the structure of group A and B samples on one side, and group C samples on the other. The cross-sectional area of a sample from each group is depicted in Figure 10

Aside from small defects arising from the printing process, the structure of group A and B samples appears homogenous with no indication of a layer-wise build-up. In group C samples, single printing layers are visible. 

**Figure 11:**
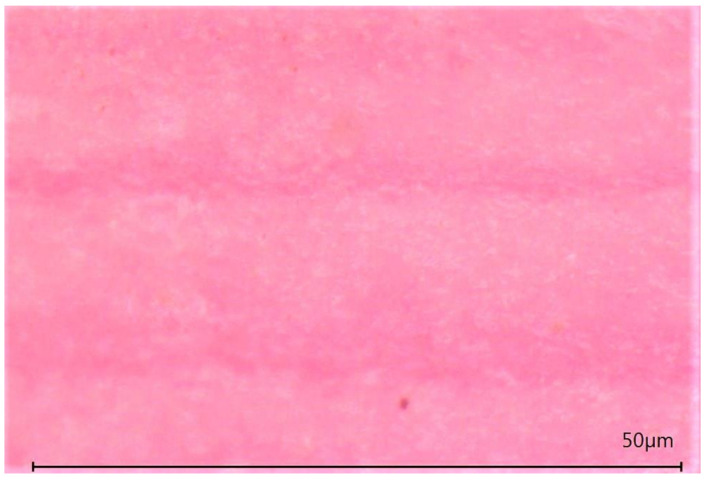
Cross-sectional area of a group C sample. Ink layers appear as thin dark lines between brighter resin layers. The specimen was colored prior to microscope imaging.

The cross-section of a group C sample is depicted in Figure 11. Thin darker lines represent ink layers while resin layers appear in brighter color. 

Microscope imaging reveals optical differences between layers in group C samples. Width of both brighter and darker bands seem to vary through the cross-section of the sample. All layers appear to be colored to a certain degree although a coloring agent is only present in ink layers during the printing process.

**Figure 12:**
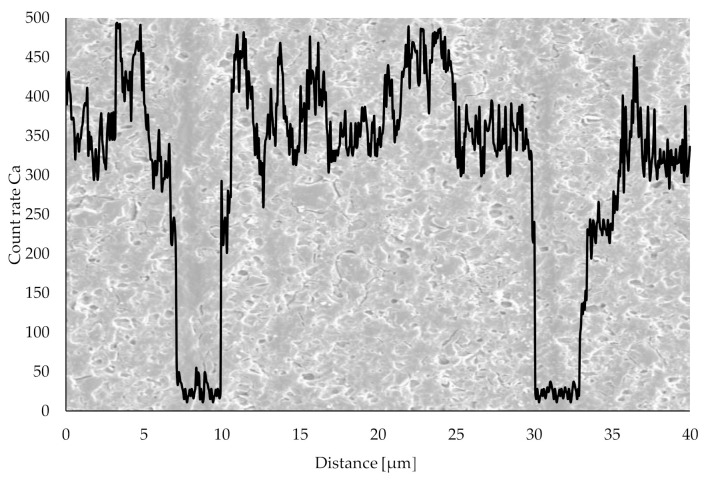
SEM image of a group C sample. Calcium content analyzed with EDX decreases significantly at the location of ink layer.

Results of the SEM analysis in combination with EDX of a group C specimen are depicted in Figure 12. The vertical axis represents the count rate for calcium characteristic for the resin. A significant decrease in calcium content at the location of ink layer not containing any TCP powder is clearly visible.

## 4. Discussion

The main aim of this bio-inspired building approach is to increase fracture toughness of an inherently brittle material with minimal alteration of other material properties. Dynstat impact strength tests reveal a significant increase of about 70% in impact strength for samples of group C. These results correspond with the bending tests, showing an increase in elongation at break of about 22%. The latter indicates a more compliant material behavior which is beneficial for fracture toughness. However, the introduction of compliant ink layers decreases Young’s modulus by almost 50% and yield strength by about 12%, thus leading to reduced strength and stiffness. All tests conducted show very similar results for groups A and B. This is remarkable taking into consideration that the same amount of ink is used in groups B and C. In a blend of mainly hard and some soft material, the latter has virtually no effect on the mechanical properties of the manufactured part. Thus, the essential role of different materials being located in separated layers is verified by comparison of groups A and B. 

The analysis of DMA curves reveals that the introduction of separate ink layers does not alter the overall temperature dependent behavior significantly. Shapes of storage modulus and tan δ curves are similar for all three groups. Still, some differences are visible. The temperature resistance of group C samples decreases. The maximum of tan δ, corresponding to the glass transition temperature, is at 83 °C and at 86 °C for groups A and B, respectively. The maximum of the tan δ curve for group C samples is at 62 °C. However, in the temperature range between 20 and 70 °C, the tan δ curve of group C is clearly above those of the other two groups. This indicates more energy dissipation inside group C samples resulting in higher fracture toughness. Storage modulus curves show similar values for all three sample groups. At room temperature (i.e., 25 °C), the values are 760 MPa, 729 MPa, and 728 MPa for groups A, B, and C, respectively. Since the storage modulus represents the elastic portion, it is connected to Young’s modulus. Therefore, the DMA measurement yields different results than bending tests conducted. The latter showing a significant decrease in Young’s modulus for group C samples compared to the other two groups (i.e., 353. 00 MPa for group A, 347.25 MPa for group B, and 175.00 for group C). However, both storage modulus and loss modulus are in frequency domain and do not have direct correlation with the elastic modulus in the time domain [57]. For a direct comparison between DMA and bending test results, additional measurements (i.e., temperature and frequency sweeps) as well as extensive transformations are required. Machines required for these measurements were not available for the authors. In the present study, microscopic architecture, interface zones, and temperature treatment might influence DMA sample properties.

Structural analysis conducted with digital microscopy imaging and SEM in combination with EDX reveals differences between groups A and B on one side and group C on the other. Layer-wise build-up is only visible in group C samples. In digital microscopy imaging, red-colored ink layers are visible as darker lines in group C samples. Nevertheless, both resin and ink layers appear to be colored to some degree. This indicates some form of diffusion process between the layers after the printing process. After the manufacturing process, the red coloring agent used as an ink component diffuses through the interfaces into other compartments. Thus, red color spreads into originally white resin layers via diffusion. Consequently, all areas in these samples are colored to some extent. Although this diffusion mechanism is neither intended nor desirable, microscopy images confirm the existence of separate layers in group C specimens. Albeit the diffusion of coloring agent discrete areas with strongly varying TCP powder content are confirmed with SEM in combination with EDX. Thus, mechanical properties strongly influenced by the TCP content vary significantly between those areas. This is the determining factor for different mechanical properties of groups B and C measured in impact and bending tests.

In this study, a completely new printing device is used to mimic the well-known bio-inspired concept of various material laminates. The hybrid printing system allows the combination of two entirely different 3D-building mechanisms (i.e., SLA and inkjet printing) to manufacture laminates with continuous alteration of materials, granting exceptional spatial resolution. This manufacturing technique allows for the processing of highly filled and high viscous liquid resins through SLA while granting the advantage of selective material placement via inkjet technology. The present manufacturing process offers a different approach towards toughening than other techniques commonly used in SLA. The latter include the adaptation of the resin via high molecular weight monomers, core-shell particles, or liquid rubbers [32]. These strategies yield an overall increase in fracture toughness; however, the toughness components are mixed into the liquid resin before the solidification. Thus, these substances exhibit a statistical distribution in the matrix. In contrast, the hybrid printing system allows for selective material placement of the toughening agent (i.e., ink) through inkjet printing. Thus, the engineering parts’ material properties can be manipulated at arbitrary locations instead of an alteration over the entire part. Although results of this investigation are promising and correspond with initial theoretical assumptions, adaptations and alterations in diverse aspects seem necessary to gain further knowledge and correct deficiencies. In this analysis, every other material layer is manufactured with ink in group C samples. Other strategies in the manufacturing process (e.g., every fifth layer is built of ink, reduced amount of ink per layer) might yield a better trade-off between stiffness and toughness that is a smaller decrease in Young’s modulus while conserving the significant increase in fracture toughness. Moreover, diffusion processes through the interfaces into other regions should be suppressed through modification of resin and ink components while maintaining sufficiently low component viscosity. Appropriate resin viscosity is a fundamental part in 3D-printing, allowing smooth material coating and constant layer-thickness. However, due to limited heating capability of the hybrid printing system and limited experience with this new 3D-printing technology, a well-approved material combination was used in this study to reduce potential sources of error during the printing process. The experiments done and results achieved in this study represent a starting point for further investigations in the future. 

## Figures and Tables

**Table 1:** Composition of photocurable resin and ink components.

Resin		Ink	
Component	Content	Component	Content
Propoxylated trimethylolpropane triacrylate (Arkema, Colombes, France)	8.72 wt. % (20.30 vol. %)	Cyanoacrylate (Abcr, Karlsruhe, Germany)	90.69 wt. % (96.89 vol. %)
Urethane dimethacrylate (Merck, Darmstadt, Germany)	7.80 wt. % (13.71 vol. %)	Poly(propylene glycol) diacrylate (Merck, Darmstadt, Germany)	6.11 wt. % (1.91 vol. %)
Poly(propylene glycol) (Merck, Darmstadt, Germany)	10.52 wt. % (16.18 vol. %)	Rhodamine B (Merck, Darmstadt, Germany)	0.12 wt. % (0.25 vol. %)
Polymeric dispersant (Lubrizol, Wickliffe, OH, USA)	0.91 wt. % (1.49 vol. %)	Phenylbis (2,4,6-trimethylbenzoyl)-phosphine oxide (BAPO) (Merck, Darmstadt, Germany)	3.08 wt. % (0.95 vol. %)
Silica (Merck, Darmstadt, Germany)	0.36 wt. % (3.73 vol. %)		
2,2-Dihydroxy-4,4-dimethoxybenzophenone (TCI, Tokyo, Japan)	0.01 wt. % (0.03 vol. %)		
(2,4,6-trimethylbenzoyl)-phenylethoxyphosphine oxide (TPO-L) (Lambson, Wetherby, UK)	0.04 wt. % (0.07 vol. %)		
β-Tricalcium phosphate (Merck, Darmstadt, Germany)	71.64 wt. % (44.48 vol. %)		

**Table 2:** Compositions and systems used for the manufacturing of each testing group.

Group	Composition	Manufacturing System
A	Resin	DLP
B	Resin and ink (blended)	DLP
C	Resin and ink (hybrid structure)	DLP and inkjet system

**Table 3:** Dimensions, number of layers, and calculated layer thickness for all test specimens.

Group	Sample	Length [mm]	Width [mm]	Height [mm]	Layers DLP	Layer Thickness DLP [µm]	Layers Inkjet	Layer Thickness Inkjet [µm]
A	Dynstat	15	10	4	160	25	0	0
A	Bending	40	25	2	80	25	0	0
A	DMA	25	4	2	80	25	0	0
B	Dynstat	15	10	4	160	25	0	0
B	Bending	40	25	2	80	25	0	0
B	DMA	25	4	2	80	25	0	0
C	Dynstat	15	10	4	160	18	160	7
C	Bending	40	25	2	80	18	80	7
C	DMA	25	4	2	80	18	80	7

**Table 4:** Results of the bending tests.

Parameter	Group A	Group B	Group C
Young’s modulus [MPa]	353. 00 ± 33.91	347.25 ± 39.48	175.00 ± 38.41
Yield strength [MPa]	11.18 ± 0.91	10.16 ± 0.19	9.81 ± 0.21
Elongation at break [%]	3.32 ± 0.24	3.10 ± 0.24	4.05 ± 0.45
